# Co-Expression Network Analysis Identifies Molecular Determinants of Loneliness Associated with Neuropsychiatric and Neurodegenerative Diseases

**DOI:** 10.3390/ijms24065909

**Published:** 2023-03-21

**Authors:** Jose A. Santiago, James P. Quinn, Judith A. Potashkin

**Affiliations:** 1 NeuroHub Analytics, LLC, Chicago, IL 60605, USA; 2 Q Regulating Systems, LLC, Gurnee, IL 60031, USA; 3 Center for Neurodegenerative Diseases and Therapeutics, Cellular and Molecular Pharmacology Department, The Chicago Medical School, Rosalind Franklin University of Medicine and Science, North Chicago, IL 60064, USA

**Keywords:** loneliness, network analysis, neurodegenerative disease, neuropsychiatric diseases, social isolation, Alzheimer’s disease, Parkinson’s disease, major depressive disorder, schizophrenia

## Abstract

Loneliness and social isolation are detrimental to mental health and may lead to cognitive impairment and neurodegeneration. Although several molecular signatures of loneliness have been identified, the molecular mechanisms by which loneliness impacts the brain remain elusive. Here, we performed a bioinformatics approach to untangle the molecular underpinnings associated with loneliness. Co-expression network analysis identified molecular ‘switches’ responsible for dramatic transcriptional changes in the nucleus accumbens of individuals with known loneliness. Loneliness-related switch genes were enriched in cell cycle, cancer, TGF-β, FOXO, and PI3K-AKT signaling pathways. Analysis stratified by sex identified switch genes in males with chronic loneliness. Male-specific switch genes were enriched in infection, innate immunity, and cancer-related pathways. Correlation analysis revealed that loneliness-related switch genes significantly overlapped with 82% and 68% of human studies on Alzheimer’s (AD) and Parkinson’s diseases (PD), respectively, in gene expression databases. Loneliness-related switch genes, *BCAM*, *NECTIN2*, *NPAS3*, *RBM38*, *PELI1*, *DPP10*, and *ASGR2,* have been identified as genetic risk factors for AD. Likewise, switch genes *HLA-DRB5*, *ALDOA*, and *GPNMB* are known genetic loci in PD. Similarly, loneliness-related switch genes overlapped in 70% and 64% of human studies on major depressive disorder and schizophrenia, respectively. Nine switch genes, *HLA-DRB5*, *ARHGAP15*, *COL4A1*, *RBM38*, *DMD*, *LGALS3BP*, *WSCD2*, *CYTH4*, and *CNTRL*, overlapped with known genetic variants in depression. Seven switch genes, *NPAS3*, *ARHGAP15*, *LGALS3BP*, *DPP10*, *SMYD3*, *CPXCR1*, and *HLA-DRB5* were associated with known risk factors for schizophrenia. Collectively, we identified molecular determinants of loneliness and dysregulated pathways in the brain of non-demented adults. The association of switch genes with known risk factors for neuropsychiatric and neurodegenerative diseases provides a molecular explanation for the observed prevalence of these diseases among lonely individuals.

## 1. Introduction

Physical distancing and social isolation measures implemented during the COVID-19 pandemic had detrimental consequences on the physical and mental health of individuals of all ages. Lack of social interactions and support can directly impact someone’s ability to cope successfully with stressful events and adapt to changes during difficult times. Loneliness, defined as the subjective perception of social isolation, is associated with a decline in physical and mental health [1]. Loneliness has been associated with numerous conditions, including major depressive disorder, anxiety, suicidal ideation, cognitive impairment, and dementia [2,3]. With the emerging increase in disease outbreaks and consequent social lockdowns, it is imperative to understand the biological and molecular mechanisms associated with loneliness and social isolation.

Several investigations have explored the neurobiological mechanisms underlying loneliness [4]. Loneliness is associated with altered structure and function in different brain regions, including the prefrontal cortex, insula, amygdala, hippocampus, and ventral striatum [4]. Transcriptomic studies in blood and brain regions have begun to unravel some of the biological and molecular mechanisms involved in loneliness and social isolation. For example, a blood transcriptomic analysis from subjects who experienced chronically high levels of social isolation identified the upregulation of genes involved in immune activation and downregulation of genes related to B lymphocyte function and type I interferon response [5]. The same group of investigators found that loneliness-induced gene expression in blood was derived primarily from antigen-presenting cells [6].

In contrast with blood, an analysis of genome-wide RNA expression in the nucleus accumbens from donors with known loneliness identified differentially expressed genes associated with behavioral processes, Alzheimer’s disease (AD), psychological disorders, cancer, and skeletal and muscular disorders [7]. The nucleus accumbens is a brain region of interest due to its involvement in reward processing and cooperative social behavior [8,9]. Likewise, loneliness-induced gene expression patterns in the dorsolateral prefrontal cortex were associated with AD, psychiatric diseases, immune dysfunction, and cancer [1].

One successful approach to investigating phenotypic transitions between healthy and disease states is the analysis of co-expression networks [10,11]. The two most common and reliable methods for constructing gene expression networks are Weighted Gene Correlation Network Analysis (WGCNA) and SWItchMiner (SWIM) [10,12]. While both approaches use a correlation matrix to construct a gene–gene similarity network, WGCNA considers only the positive correlation between gene pairs. In contrast, one strength of the SWIM method is the consideration of the negative correlation of the correlation distribution. The emphasis on the left tail allows the identification of genetic drivers called ‘switch genes,’ which are anticorrelated with their neighbors in the correlation network. In other words, when switch genes are induced, their interaction partners are repressed and vice versa. These advantages have been explained in detail in ref. [13]. One limitation of this approach is that it is based on correlations; thus, causal relationships cannot be conclusively established.

Switch genes are molecular drivers responsible for drastic transcriptional changes involved in phenotypic transitions. This network method has been instrumental in the identification of switch genes in AD, vascular dementia, frontotemporal dementia (FTD), amyotrophic lateral sclerosis (ALS), physical activity, and cancer [14,15,16,17,18,19,20]. Here, we implemented a bioinformatics approach including co-expression networks and comparative transcriptomic analyses to characterize the molecular pathways involved in loneliness and social isolation. We present evidence that molecular determinants of loneliness are intimately related to neurodegenerative and neuropsychiatric diseases.

## 2. Results

### 2.1. Database Mining and Study Selection

**Figure 1:**
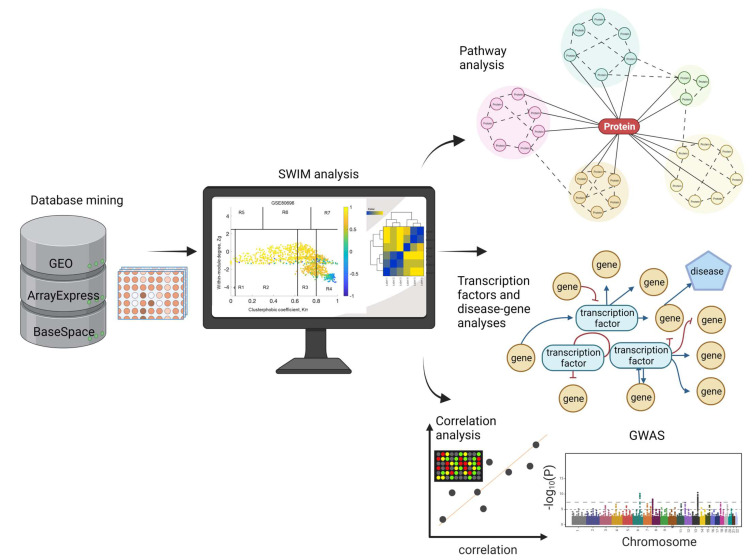
Overall bioinformatics workflow. We searched the GEO, ArrayExpress, and BaseSpace Correlation Engine to identify RNA expression studies on loneliness or social isolation. The arrays that met our inclusion/exclusion criteria were analyzed further using SWIM analysis, pathways, disease–gene networks, and transcription factors. Finally, we performed correlation analyses in BaseSpace between gene expression patterns induced by loneliness and neuropsychiatric and neurodegenerative diseases. We identified shared genetic risk factors between loneliness and neuropsychiatric and neurodegenerative diseases.

We searched the Gene Expression Omnibus (GEO), BaseSpace Correlation Engine (BSCE, Illumina, Inc, San Diego, CA, USA), and ArrayExpress databases to identify microarrays from subjects with known loneliness (See Section 4). The following arrays were retrieved on 21 July 2022: GEO = 397, ArrayExpress = 6, BSCE = 6. One array, GSE80696, which contained transcriptomic data from individuals with known loneliness, met our inclusion/exclusion criteria and was analyzed further. This dataset can be accessed using the GEO database link: https://www.ncbi.nlm.nih.gov/geo/query/acc.cgi?acc=GSE80696, 21 July 2022. The overall bioinformatics workflow is presented in Figure 1.

### 2.2. Identification of Switch Genes Associated with Loneliness

The dataset GSE80696 was imported into SWItchMiner (SWIM) to identify switch genes. The SWIM analysis was performed using the following comparisons: all subjects (high vs. low loneliness), males (high vs. low loneliness), and females (high vs. low loneliness).

**Figure 2:**
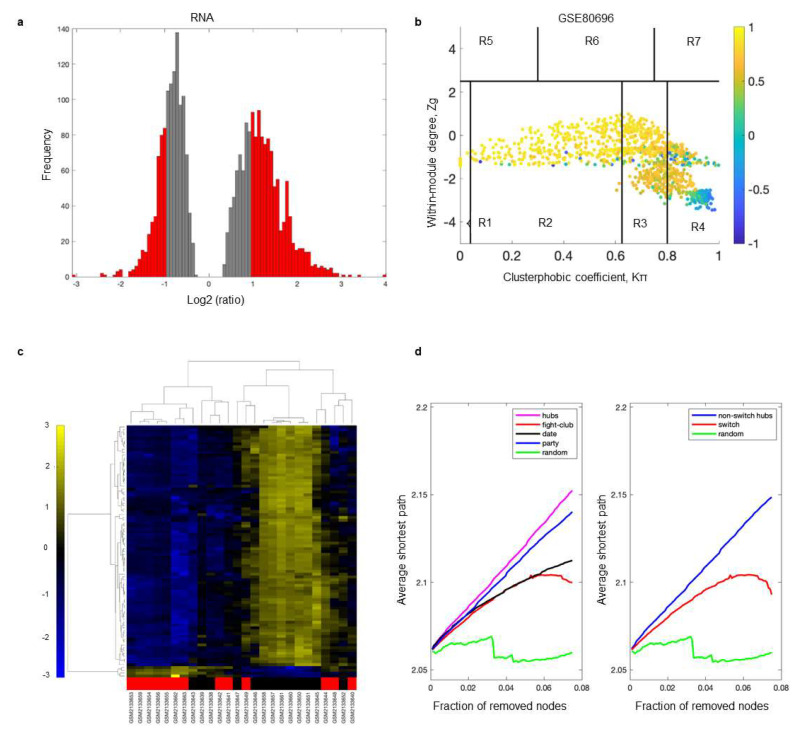
Identification of switch genes. SWIM analysis of postmortem nucleus accumbens of subjects with chronic loneliness compared to those with low loneliness (GSE80696). (**a**) Distribution of log2 fold change values where the red bars were selected for further analysis. (**b**) Heat cartography map with nodes colored by their average Pearson’s correlation coefficient. Blue nodes in region R4 represent switch genes. (**c**) Dendrogram and heatmap for switch genes. The red markers indicate samples from subjects with chronic loneliness. (**d**) Robustness of the correlation network.

The SWIM algorithm was performed as previously described [10,20,21]. First, genes were included (red bars) or discarded (gray bars) using a cut-off of 2.0 or higher (Figure 2a). The gene matrix was imported into SWIM to build the loneliness gene correlation network based on the average Pearson correlation coefficient (APCC). Using the APCC, three hubs were defined; date hubs with low positive co-expression with their partners, party hubs with high positive co-expression, and fight-club hubs with negative APCC values (Figure 2b). Two parameters identified the plane, *Zg* (within-module degree) and *Kπ* (clusterphobic coefficient), which was divided into seven regions, each defining a specific node role (R1-R7). High *Zg* values corresponded to hub nodes within their module (local hubs), whereas low *Zg* values corresponded to nodes with few connections within their module (non-hubs within their communities, but they could be hubs in the network). Each node was colored according to its APCC value. Yellow nodes were party and date hubs, which were positively correlated in expression with their interaction partners. The switch genes were characterized by low *Zg* and high *Kπ* values and were connected mainly outside their module. The switch genes were the blue nodes in region R4 (Figure 2b).

An expression heatmap of switch genes is presented in Figure 2c. The data were clustered according to rows and columns representing switch genes and samples, respectively. The samples depicted in red were from subjects with high loneliness. Most switch genes identified in individuals with high loneliness were downregulated (shown in blue). In contrast, those with low loneliness were upregulated (shown in yellow) (Figure 2c). Fight-club hubs differed from the date and party hubs, and switch genes were significantly different from random genes, confirming the analysis’s robustness (Figure 2d). The *x*-axis represented the cumulative fraction of removed nodes. In contrast, the *y*-axis represented the average shortest path. Each curve corresponded to the variation in the average shortest path of the correlation network as a function of removing nodes specified by the colors of each line.

SWIM analysis identified 48 switch genes in the nucleus accumbens from individuals with high vs. low loneliness. The same analysis was performed by stratifying the samples by sex and level of loneliness. This analysis yielded 27 switch genes in males with increased loneliness compared to low loneliness (Supplementary Figure S1). Switch genes from males with high loneliness depicted with red bars were downregulated (shown in blue) compared to males with low loneliness (Supplementary Figure S1). An analysis of samples from females did not yield any switch genes.

### 2.3. Biological and Functional Analysis of Switch Genes

Functional associations were explored using the HUGO database. Gene ontology revealed that some switch genes from individuals with high loneliness were associated with angiogenesis and hemostasis (*SERPINA1*, *FN1*, *KLK3*, *COL4A1*), innate immunity and inflammation (*CD59*, *GPNMB*, *LILRA2*, *NECTIN2*, *UBE2V1*), lipid metabolism (*GPR3*, *SRD5A1*, *ACSL5*, *ACACB*), and neuronal development and function (*ARF1*, *FN1*, *DPP10*, *DMD*, *TH*, *SYT8*, *FOXN4*). In males with high loneliness, switch genes were involved in the inflammatory response (*HAMP*, *ZFP36*, *PELI1*, *CXCL1*, *HLA-DRB5*, *S100A8*, *SPN*) and regulation of transcription (*NPAS3*, *AGO2*, *FOS*, *TAF6*). The list of switch genes and their gene ontology annotations is provided in Supplementary Table S2.

**Figure 3:**
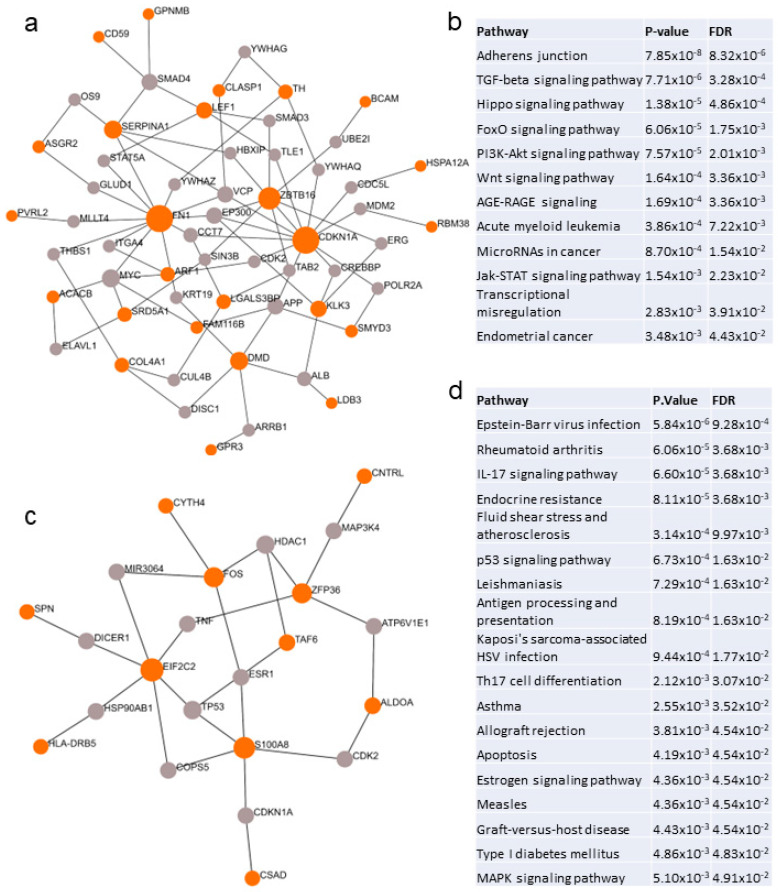
Network and pathway analysis of switch genes. (**a**) Network analysis of loneliness switch genes. Switch genes are depicted in orange and interacting proteins are in gray. (**b**) Unique pathways dysregulated in individuals with chronic loneliness. (**c**) Network analysis of switch genes identified in males with chronic loneliness. (**d**) Unique pathways dysregulated in males with chronic loneliness. Network and pathway analysis was performed in NetworkAnalyst.

Network analysis revealed 12 unique pathways associated with loneliness (Figure 3a,b, Supplementary Table S3). The top pathways implicated in loneliness were adherens junctions, TGF-β, FOXO, Hippo, PI3K-AKT, WNT, AGE-RAGE, acute myeloid leukemia, microRNAs in cancer, JAK-STAT, and endometrial cancer (Figure 3b). Network analysis identified 18 unique pathways associated with loneliness in males (Figure 3c,d, Supplementary Table S3). The male pathways were predominantly associated with infection, innate immunity, cancer-related pathways, and autoimmune diseases (Figure 3d). Venn diagram analysis indicated that 15 pathways were shared between both groups. The complete list of pathways is provided in Supplementary Table S3.

### 2.4. Gene–Disease Association Analysis

**Figure 4:**
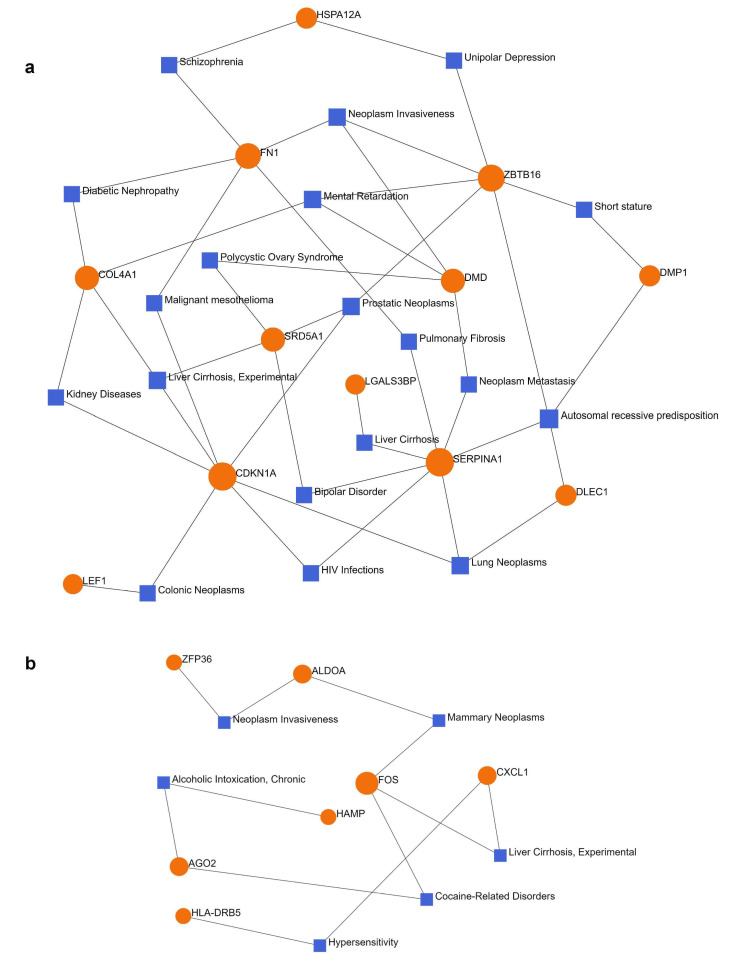
Disease–gene network analysis. (**a**) Disease–gene network analysis of switch genes from subjects with chronic loneliness and (**b**) diseases associated with switch genes from males with chronic loneliness. Switch genes are depicted in orange and diseases are in blue.

A gene–disease association network analysis was performed in NetworkAnalyst. Switch genes obtained from lonely individuals were connected to 16 diseases, including cancer, liver cirrhosis, HIV infection, bipolar disorder, depression, schizophrenia, and mental retardation (Figure 4a, Supplementary Table S4). Male-specific switch genes were connected to six diseases, including liver cirrhosis, cocaine-related disorders, alcoholic intoxication, mammary neoplasms, and hypersensitivity (Figure 4b, Supplementary Table S4).

### 2.5. Gene–Transcription Factor Network Analysis

Transcription factor analysis of loneliness-related switch genes identified 65 master regulators. The most significant transcription factors according to degree and betweenness centrality were *IRF1* and *TGIF2* (Supplementary Table S5). Analysis of loneliness switch genes from males identified 41 transcriptional regulators. The most significant transcription factors based on network topology measurements were *KLF9* and *ZFX* (Supplementary Table S5).

The lists of transcription factors were analyzed further using the String database (https://string-db.org/, accessed on 1 September 2022). Analysis of the list of transcription factors from individuals with chronic loneliness compared to low loneliness identified 195 biological processes. The most significant processes were associated with the negative regulation of transcription, RNA metabolic process, nucleobase-containing compound metabolic process, and cellular macromolecule biosynthetic process. Transcription factors were related to zinc finger protein domains C2H2 type. In contrast, transcription factors from males with chronic loneliness were associated with the positive regulation of nucleic-acid transcription, RNA metabolic process, cellular macromolecule biosynthetic process, and cellular metabolic process. Venn diagram analysis showed that TFs from males with chronic loneliness were enriched in 12 unique biological pathways, including positive regulation of RNA metabolism, regulation of erythrocyte differentiation, lymphocyte differentiation, response to alcohol, leukocyte activation, leukocyte differentiation, cellular response to IL-6, positive regulation of pri miRNA transcription, cellular response to IL-7, erythrocyte differentiation, cellular response to oxygen-containing compound, and response to lipopolysaccharide (Supplementary Table S5).

### 2.6. Loneliness-Related Switch Genes Associated with Neuropsychiatric and Neurodegenerative Diseases

We investigated whether loneliness-related switch genes were involved in neuropsychiatric and neurodegenerative diseases. We compared the results from this study to our previous analyses of switch genes in AD, FTD, ALS, and physical activity [14,15,19]. Several loneliness-related switch genes were identified as switch genes in different neurodegenerative diseases. For instance, *ACACB* and *DLEC1* were identified as switch genes in the entorhinal cortex of AD patients [14]. Further, *ACACB* and *GPNMB* were identified as switch genes in the frontal cortex of FTD patients [15].

We curated the literature to explore the associations between loneliness-related switch genes and brain diseases. Specifically, we used the search terms “neurodegeneration”, “dementia”, “Alzheimer’s disease”, “Parkinson’s disease”, “Frontotemporal dementia”, “Amyotrophic lateral sclerosis”, “Lewy body dementia”, “neuropsychiatric disorders”, “major depressive disorder”, and “schizophrenia” for each switch gene individually. This search identified the association of 25 switch genes with neurodegenerative and neuropsychiatric diseases. For instance, 13 switch genes, *GPNMB*, *TH*, *CD59*, *COL4A1*, *ZBTB16*, *TSPAN15*, *DMD*, *LEF1*, *GPR3*, *UBE2V1*, *DPP10*, *NECTIN2*, *LGALS3BP*, *CDKN1A*, *SERPINA1*, and *DMP1* were linked to AD, PD, FTD, PD dementia, Creutzfeldt Jakob disease, and LBD (Table 1). Nine switch genes from the male dataset, *AGO2*, *HLA-DRB5*, *ALDOA*, *S100A8*, *CTSG*, *CXCL1*, *CYTH4*, *PELI1*, and *FPR1,* were associated with AD, HD, PD, LBD, and FTD. Five switch genes, *HLA-DRB5*, *CYTH4*, *NPAS3*, *DMD*, and *DPP10,* were related to neuropsychiatric disorders (Table 1).

### 2.7. Comparative Gene Correlation Analysis between Loneliness and Neuropsychiatric and Neurodegenerative Diseases

Given the associations between loneliness and brain diseases, we performed a correlation analysis between the switch genes identified from chronically lonely subjects (GSE80696) and the most common neuropsychiatric and neurodegenerative diseases using the BSCE database. We used the search terms “Alzheimer’s disease”, “dementia”, “Parkinson’s disease”, “major depressive disorder”, “depression”, and “schizophrenia” to identify arrays. Studies were filtered only to include human studies.

The correlation analysis showed that loneliness-related switch genes overlapped in 82% (53/65) of human studies on AD deposited in the BSCE database (Table S6). The most significant genetic overlap was observed in studies of entorhinal and frontal cortex pyramidal neurons from early-stage AD patients. Specifically, 23 (*p* = 1.60 × 10^−9^) and 36 (6.10 × 10^−7^) switch genes overlapped in the entorhinal and frontal cortices, respectively. Furthermore, several loneliness-related switch genes were associated with variants previously identified as risk factors for AD in 30 different GWAS studies (Supplementary Table S7). For instance, variants in *BCAM* and *NECTIN2* have been related to the risk of AD in more than 10 GWAS from diverse populations, including European, Caucasian, and Japanese (Supplementary Table S7). *NPAS3*, *RBM38*, and *PELI1* were associated with the risk of AD in *APOE4* (-) individuals and AD with psychosis in a European cohort. Finally, *ASGR2* was associated with the response to cholinesterase inhibitors in discovery and replication cohorts of AD individuals (Supplementary Table S7).

The same analysis was performed with PD studies. Loneliness-related switch genes overlapped in 68% (40/59) of human studies on PD (Table S8). The most significant genetic overlap was observed in the globus pallidus internal of PD patients with 12 overlapping switch genes (*p* = 2.50 × 10^−6^). Several switch genes overlapped with known risk loci in PD patients. For example, variants in *NPAS3*, *HLADRB5*, *ALDOA*, and *GPNMB* have been linked to PD risk in several populations (Supplementary Table S9).

In the context of neuropsychiatric diseases, loneliness switch genes overlapped in 70% (16/23) of human studies on major depressive disorder (Supplementary Table S10). Nine switch genes, *HLA-DRB5*, *ARHGAP15*, *COL4A1*, *RBM38*, *DMD*, *LGALS3BP*, *WSCD2*, *CYTH4*, and *CNTRL*, overlapped with known genetic variants in depression (Supplementary Table S11). Similarly, switch genes overlapped in 64% (16/25) of human studies in schizophrenia (Supplementary Table S12). Seven switch genes, *NPAS3*, *ARHGAP15*, *LGALS3BP*, *DPP10*, *SMYD3*, *CPXCR1*, and *HLA-DRB5,* were associated with known risk factors for schizophrenia (Supplementary Table S13).

## 3. Discussion

We performed a bioinformatics approach to identify genes responsible for drastic transcriptional changes occurring in the brain of individuals exposed to chronic levels of loneliness. Co-expression network analysis using SWIM identified 48 switch genes in the postmortem nucleus accumbens from individuals with high loneliness compared to low loneliness. Analysis stratified by sex identified 27 switch genes in males with chronic loneliness.

Network analysis of loneliness-related switch genes revealed enrichment in several unique pathways, including adherens junction, TGF-β, Hippo, FOXO, PI3K-AKT, WNT, JAK-STAT, AGE-RAGE signaling in diabetic complications, and cancers. Among these pathways, TGF-β has been implicated in the pathogenesis of neuropsychiatric mood disorders and neurodegeneration due to its central actions in regulating the stress response [69]. For example, deficient TGF-β signaling triggered neurodegeneration by promoting amyloid β accumulation and dendritic loss in a mouse model of AD [70]. Likewise, FOXO and PI3K-AKT have been implicated in neurodegeneration. FOXO1 and genes under its regulation have been implicated in the pathogenesis of PD [71]. FOXO and PI3K-AKT signaling are involved in lipid metabolism and insulin signaling and may be shared pathways between diabetes and AD [72,73,74]. Similarly, advanced glycation end products (AGE) and their receptor RAGE may contribute to or protect against AD by regulating inflammatory mechanisms [75].

Transcription factor analysis identified *IRF1* and *TGFI2* as the most significant regulators of loneliness switch genes. *IRF1* plays a role in immunity, anti-viral mechanisms, macrophage polarization, and microglial activation [76,77,78]. Interestingly, *IRF1* is regulated by *BIN*, the second most common risk factor for AD, and has essential roles in regulating the brain inflammatory response and microglial function [79,80]. TGFI2 is associated with neuronal apoptosis, neocortical development, neurogenesis, brain defects, and mental retardation [81,82].

In contrast, switch genes from lonely males were enriched predominantly in infection, autoimmune diseases, and antigen processing and presentation. These findings were consistent with previous work in which antigen-presenting cells in blood were identified as the primary targets and most transcriptionally sensitive immune cells to social isolation [6]. Genetic changes associated with loneliness were derived primarily from plasmacytoid dendritic cells, monocytes, and B cells. Furthermore, several immune-related switch genes, including *HLA-DRB5*, *CXCL1*, and *PELI1*, were exclusively identified in males exposed to chronic isolation.

Network analysis of transcription factors identified *KLF9* as the most significant regulator of loneliness-related switch genes in males. *KLF9* has been identified as an important transcriptional regulator in the hippocampus of AD patients [14]. Furthermore, *KLF9* promotes the expression of *PGC1α*, a critical factor in hepatic gluconeogenesis [83], mitochondrial function, and a potential therapeutic target in PD [84,85].

Biological and functional analyses revealed interesting differences in pathways regulated by transcription factors identified from males with chronic loneliness and those from all subjects. Transcription factors obtained from all subjects were enriched primarily in the negative regulation of both transcription and RNA metabolism. In contrast, transcription factors from males with chronic loneliness were involved in the positive regulation of both transcription and RNA metabolism. In this regard, disrupted RNA metabolism and processing has been recognized as a critical determinant in neurological diseases including ALS, AD, FTD, and PD [86,87,88]. Moreover, transcription factors were enriched in chromatin organization and C2H2 zinc finger protein, which are involved in chromatin closing [89].

Interestingly, transcription factors from males were uniquely enriched in pathways related to the response to alcohol, leukocyte differentiation, and the cellular response to IL-6, IL-7, and lipopolysaccharide. These findings reinforce the involvement of alcohol addiction and innate immunity in males with chronic loneliness.

Together, these findings suggest that loneliness directs transcriptional changes that influence the dysregulation of pathways involved in lipid metabolism, insulin signaling, RNA metabolism, and inflammatory processes. Given the evidence from blood and brain studies, it is plausible to speculate that pathways related to innate immunity are predominantly disrupted in males exposed to chronic loneliness.

We next investigated the linkage between loneliness-related switch genes and other diseases. Disease–gene network analysis revealed that chronic loneliness switch genes were associated with various cancers, liver cirrhosis, and neuropsychiatric conditions, including mental retardation, depression, bipolar disorder, and schizophrenia. Switch genes identified in males exposed to chronic loneliness were linked to cocaine addiction, mammary neoplasms, hypersensitivity, and alcoholic intoxication. These findings suggest that loneliness induces the transcription of genes associated with malignancies and neuropsychiatric conditions. Males exposed to chronic loneliness may be more prone to alcohol use, cocaine addiction, and infection. For example, depressed men reported higher rates of anger attacks, aggression, substance abuse, and risk-taking than women [90]. Moreover, male-specific transcriptional rewiring of genes involved in alcohol and cocaine addiction has been recently identified in AD patients ([91]).

Loneliness has been documented to promote cognitive decline and neurodegeneration, but the specific molecular determinants underlying this association are unclear. We investigated how loneliness-related switch genes are associated with neurodegenerative and neuropsychiatric diseases. Interestingly, manual curation of the literature revealed that 25 loneliness-related switch genes had been implicated in various neurodegenerative and neuropsychiatric disorders, including AD, PD, HD, FTD, depression, and schizophrenia.

Correlation analysis showed that loneliness-related switch genes overlapped with 82% of human gene expression studies in AD deposited in the BSCE database. Notably, several switch genes are associated with risk variants for AD. Loneliness-related switch genes *BCAM*, *NECTIN2*, *NPAS3*, *RBM38*, *PELI1*, *DPP10*, and *ASGR2* were previously identified as risk factors for AD in several populations [92,93,94,95,96,97,98,99,100].

Furthermore, loneliness switch genes significantly overlapped with 68% of human gene expression studies in PD. Similar to AD, several switch genes are associated with known genetic loci in PD. Mutations in *NPAS3*, *HLA-DRB5*, *ALDOA*, and *GPNMB* have been associated with PD risk in several GWAS [101,102,103,104]. These findings suggest that loneliness induces drastic gene expression changes consistent with a neurodegeneration phenotype. Individuals exposed to chronic loneliness may be more susceptible to AD or PD, possibly through different pathways.

Several studies have indicated a linkage between loneliness and neuropsychiatric diseases. A population-based study reported that higher loneliness scores were associated with higher depression symptom severity [105]. In this study, loneliness-related switch genes significantly overlapped with 70% and 64% of human gene expression studies in major depressive disorder and schizophrenia, respectively. Several loneliness switch genes have been reported as genetic risk factors for these diseases. For example, *HLA-DRB5*, *ARHGAP15*, *COL4A1*, *RBM38*, *DMD*, *LGALS3BP*, *WSCD2*, *CYTH4*, and *CNTRL* overlapped with known genetic variants in depression, reinforcing the idea that loneliness may increase disease susceptibility [106,107,108]. Likewise, *NPAS3*, *ARHGAP15*, *LGALS3BP*, *DPP10*, *SMYD3*, *CPXCR1*, and *HLA-DRB5* were associated with known risk factors for schizophrenia [97,109,110,111,112]. In this regard, social isolation during the pandemic correlated with paranoid ideation [113]. A genetic variant near the switch gene CPXCR1 was associated with schizophrenia risk in Japanese males in a replication cohort [114].

Sex-specific differences in symptoms and conditions have been noted among lonely individuals. For example, loneliness was associated with major depressive disorder and anxiety, especially in men, during the COVID-19 pandemic [115]. Specifically, men reported higher rates of depressive symptoms and suicidal ideation than women during the COVID-19 pandemic [116]. Symptoms of depression in males may be different from those observed in females. In this regard, when male-type symptoms of depression are included in depression rating scales, a higher proportion of males than females met the criteria for depression [90]. Identifying loneliness-related switch genes in males but not females may suggest that loneliness may have a more drastic transcriptional impact in the brain of males, making them more susceptible to some neurodegenerative and neuropsychiatric disorders. Future longitudinal and sex-stratified studies are needed to understand how loneliness impacts the brain of males and females differently.

Lifestyle changes may be useful strategies to mitigate the negative effects of loneliness in older adults. Physical activity, for example, has been shown to promote synaptic growth and reduce inflammation, thus protecting the brain against oxidative stress and neurodegeneration [19]. Other lifestyle modifications, including diet, sleep hygiene, mindfulness, and meditation, have been proposed to benefit the brain against depression, cognitive decline, and neurodegeneration [117,118,119,120].

**Figure 5:**
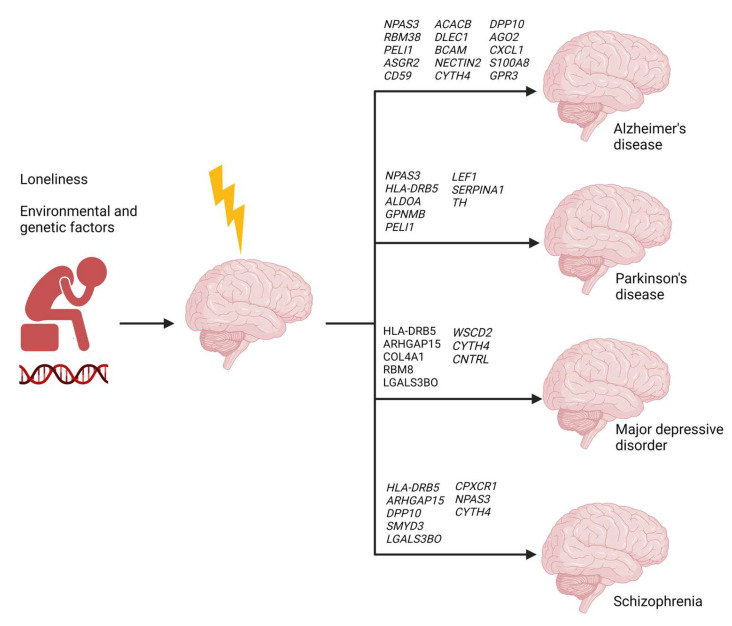
The impact of loneliness on brain health. Loneliness, social isolation, environmental stressors, and genetic factors can have detrimental effects on the brain and may lead to several neuropsychiatric and neurodegenerative diseases. Several switch genes responsible for the dramatic transcriptional changes in the brains of lonely individuals have been reported to play a role in the pathogenesis of neuropsychiatric and neurodegenerative diseases.

Several limitations are noteworthy. The findings presented herein are derived from bioinformatics analyses. Further mechanistic studies are needed to confirm the functional role of these switch genes. Validation of these results in an independent human gene expression dataset will be critical to determine the reproducibility of these findings in other patient populations. The study GSE80696 contained transcriptomic data from White, non-Hispanic individuals; thus, the switch gene analysis is not representative of the overall population. Notably, lonely individuals in this cohort showed poorer cognitive function than non-lonely subjects; therefore, correlations between loneliness-related switch genes and neurodegeneration are not unexpected. Nonetheless, the findings presented herein provide evidence that loneliness, in combination with environmental and genetic factors, induces gene expression changes in the brain that may lead to the development of several neuropsychiatric and neurodegenerative diseases (Figure 5). The association of switch genes with known risk factors for neuropsychiatric and neurodegenerative diseases provides supporting molecular evidence for the observed prevalence of these diseases among lonely individuals. Future longitudinal studies on loneliness will be crucial for a better understanding of the impact of loneliness on brain health.

## 4. Materials and Methods

### 4.1. Microarray Dataset Selection

We searched the GEO (https://www.ncbi.nlm.nih.gov/gds, accessed on 21 July 2022), BSCE, and ArrayExpress databases in August 2022 for transcriptomic studies using the search terms “homo sapiens”, “human”, “loneliness”, and “social isolation.” The inclusion criteria were: (1) human microarrays from relevant tissues in loneliness or social isolation, (2) 3 samples or more. The exclusion criteria were: (1) animal and cellular models. One dataset met our criteria and was processed for SWIM and pathway analyses. The dataset GSE80696 included postmortem transcriptomic data from the nucleus accumbens from 26 White, non-Hispanic subjects without known dementia and depression at enrollment in the Rush Memory and Aging Project (MAP) [7]. These participants were selected from a cohort of 247 MAP participants with reported loneliness scores. The clinical characteristics of the study participants in GSE80696 are described in detail elsewhere [7] and in Supplementary Table S1.

### 4.2. Identification of Switch Genes

Raw data from GSE80696 were imported into SWIM to identify switch genes. The SWIM algorithm has been described in detail in ref. [10,20,21]. We performed the following comparisons: all individuals with high vs. low loneliness, and samples stratified by sex, males and females with high vs. low loneliness. Genes with no or low expression were removed in the preprocessing stage. SWIM analysis works best with a network size between 1000–2000 nodes. The fold change parameter was adjusted to optimize the network size. For comparing all subjects with high vs. low loneliness, a fold change threshold of 2.0 was used. For the sex-stratified analysis, a fold change threshold of 4 was used. These fold changes were set for each array in the filtering step, and genes that were not significantly expressed between cases compared to controls were removed. The False Discovery Rate method (FDR) was used for multiple test corrections. Pearson’s correlation test was performed to build a co-expression network of genes differentially expressed between individuals with high vs. low loneliness. The k-means algorithm was used to identify communities within the network. SWIM uses a Scree plot to determine the number of clusters, and the clusters with the lowest number of sums of the square error (SSE) values among the replicates are designated as the number of clusters. We built a heat cartography map using the clusterphobic coefficient Kπ and the global-within module degree Zg. The coefficient *Kπ* measures the external and internal node connections, whereas *Zg* measures the extent to which each node is connected to others in its community. A node was considered a hub when Zg > 5. The average Pearson’s correlation coefficient (APCC) between the expression profile of each node and its nearest neighbors was used to build the heat cartography map. Three hubs were defined; date hubs that showed low positive co-expression with their partners (low APCC), party hubs that showed high positive co-expression (high APCC), and nodes that had negative APCC values were called fight-club hubs. Switch genes interact outside their community, are not in local hubs, and are mainly anticorrelated with their interaction partners.

### 4.3. Functional Analysis of Switch Genes

Gene ontology associations were explored for each switch gene using the HUGO database (https://www.genenames.org/, accessed on 1 September 2022). For pathway analysis, official gene symbols were imported into NetworkAnalyst (https://www.networkanalyst.ca/, accessed on 1 September 2022) [121]. Tissue-specific networks were built using the nucleus accumbens protein-protein interaction database in NetworkAnalyst. The minimum connected network was selected for further pathway analysis. Data derived from KEGG were used for pathway selection. A gene–disease association network analysis was performed in NetworkAnalyst. Nodes were ranked according to network topology measures, degree, and betweenness centrality. A *p*-value and FDR of less than 0.05 were considered significant.

### 4.4. Transcription Factor Analysis

Transcription factor analysis was performed in NetworkAnalyst. The lists of switch genes obtained from subjects exposed to chronic loneliness and those obtained from males with high loneliness were analyzed separately. Transcription factor data were derived from the Encyclopedia of DNA Elements (ENCODE). ENCODE uses the BETA Minus Algorithm in which only peak intensity signal <500 and the predicted regulatory potential score <1 are used. Transcription factors were ranked according to network topology measurements, including degree and betweenness centrality. Biological and functional analysis of transcription factors was performed using the String database (https://string-db.org/, accessed on 1 September 2022). Pathways with FDR < 0.05 were denoted as significant.

### 4.5. Gene Expression and Correlation Analyses

Gene correlation analysis was performed using the curated BSCE database. The switch genes identified in subjects with chronic loneliness were compared to gene expression profiles from subjects with neuropsychiatric and neurodegenerative diseases using the correlation tool. The genetic overlap between GSE80696 and the other datasets was analyzed as previously described [19,74]. For the correlation analysis, the number of shared genes was compared between any two datasets. BSCE uses a “Running Fisher” algorithm to compute the overlapping *p*-values between different gene expression datasets [122]. Genes below the 20th percentile of the combined normalized signal intensities were removed. The scoring and ranking of a gene were calculated according to the activity of each gene in each dataset and the number of datasets in which the gene is differentially expressed. Ranks were normalized to eliminate bias owing to varying platform sizes. Only genes with a *p*-value of 0.05 or less and an absolute fold-change of 1.2 or greater were considered significant. 

## Figures and Tables

**Table 1:** Association of loneliness-related switch genes with neurodegenerative and neuropsychiatric diseases.

Switch Gene	Name	Dataset (High vs. Low Loneliness)	Neurodegenerative Diseases	Neuropsychiatric Diseases	References
AGO2	Argonaute 2	Males	Silencing of AGO2 enhances the expression of APP-cleaving enzyme (BACE1) in vitro. AGO2 accumulation leads to dysregulation of miRNAs and impairment of autophagy in Huntington’s disease.		[22,23]
HLA-DRB5	Major histocompatibility complex, class II, DR beta 5	Males	HLA-DRB5 variants are linked to neuroinflammation and AD.	HLA-DRB5 is associated with remission to antidepressant treatment and epigenetic alterations in schizophrenia.	[24,25,26,27]
ALDOA	Aldolase, fructose-bisphosphate A	Males	Increased APOE4 copy number is associated with increased ALDOA levels in CSF of AD patients.		[28]
S100A8	S100 calcium-binding protein A8	Males	Myeloperoxidase is accumulated in S100A8(+) neutrophils in the AD human brain. S100A8 is increased in the serum of FTD patients. Aggregation of S100A8 precedes Aβ formation in mice.		[29,30,31]
CTSG	Cathepsin G	Males	Dysregulated expression of CTSG is associated with Lewy body dementia.		[32]
CXCL1	C-X-C motif chemokine ligand 1	Males	Upregulated CXCL1 mediates Aβ toxicity in the human AD brain.		[33]
CYTH4	Cytohesin 4	Males	Expression levels of CYTH4 alleles are increased in AD patients.	CYTH4 variants are associated with schizophrenia in primates and bipolar disorder in humans.	[34,35]
PELI1	Pellino E3 ubiquitin-protein ligase 1	Males	PELI1 is involved in microglial activation and dopaminergic cell death in PD.		[36]
FPR1	Formyl peptide receptor 1	Males	FPR1 is associated with dementia risk.		[37,38]
NPAS3		Males		NPAS3 variants are implicated in schizophrenia and intellectual disability.	[39,40,41,42,43,44]
GPNMB	Glycoprotein nonmetastatic melanoma protein B	All	GPNMB confers risk for PD. It is increased in the plasma of PD patients and associated with disease severity. GPNMB is a switch gene in the frontal cortex of FTD patients.		[15,45]
TH	Tyrosine hydroxylase	All	A deficiency of the TH enzyme is a critical feature in PD.		[46,47]
CD59	Complement defense 59	All	CD59 deficits are observed in AD patients’ frontal cortex, hippocampus, and plasma.		[48,49]
COL4A1	Collagen type IV alpha 1 chain	All	COL4A1 mutation is associated with multi-infarct dementia.		[50]
ZBTB16	Zinc finger and BTB domain-containing 16	All	ZBTB16 is linked to increased autophagy in Huntington’s and AD mice models.		[51,52,53]
TSPAN15	Tetraspanin 15	All	TSPAN15 is increased in human and animal models of AD.		[54]
DMD	Dystrophin	All	DMD is a crucial hub gene associated with AD risk variants.	There is a higher prevalence of neuropsychiatric diseases among patients with Duchene and Becker muscular dystrophies.	[55,56]
LEF1	Lymphoid enhancer-binding factor 1	All	LEF1 is involved in the differentiation of midbrain dopaminergic neurons.		[57]
GPR3	G protein-coupled receptor 3	All	Loss of GPR3 reduced Aβ formation and improved memory in AD mouse models. Elevated GPR3 expression is correlated with AD progression in humans.		[58]
UBE2V1	Ubiquitin-conjugating enzyme E2 V1	All	UBE2V1 is downregulated in the blood of AD patients.		[59]
DPP10	Dipeptidyl peptidase like 10	All	DPP10 malfunctioning is associated with AD and FTD.	DPP10 variants are associated with bipolar disorder and schizophrenia	[60,61,62]
NECTIN2	Nectin cell adhesion molecule 2	All	NECTIN2 variants are associated with AD risk.		[63]
LGALS3BP	Galectin-3-binding protein	All	Increased secretion of GAL3BP suppressed Aβ production in a cellular model of AD.		[64]
CDKN1A	Cyclin-dependent kinase inhibitor 1A	All	CDKN1A is increased in the blood of AD patients.		[64]
SERPINA1	Serpin family A member 1	All	SERPINA1 is a risk marker for PD dementia. SERPINA1 is upregulated in CSF of Creutzfeldt-Jakob disease and FTD patients. SERPINA1 isoforms were differentially expressed in CSF of AD and LBD patients.		[65,66,67]
DMP1	Dentin matrix acidic phosphoprotein 1	All	Silencing of DMP1 improved cognitive impairment and enhanced the proliferation of neural progenitor cells in AD mice.		[68]

## Data Availability

All the data is included in the paper and supplementary material.

## Supplementary Materials

The following supporting information can be downloaded at: https://www.mdpi.com/article/10.3390/ijms24065909/s1.

## References

[1] Canli T., Yu L., Yu X., Zhao H., Fleischman D., Wilson R.S., De Jager P.L., Bennett D.A. (2018). Loneliness 5 years ante-mortem is associated with disease-related differential gene expression in postmortem dorsolateral prefrontal cortex. Transl. Psychiatry.

[2] Beutel M.E., Klein E.M., Brähler E., Reiner I., Jünger C., Michal M., Wiltink J., Wild P.S., Münzel T., Lackner K.J. (2017). Loneliness in the general population: Prevalence, determinants and relations to mental health. BMC Psychiatry.

[3] Kuiper J.S., Zuidersma M., Oude Voshaar R.C., Zuidema S.U., van den Heuvel E.R., Stolk R.P., Smidt N. (2015). Social relationships and risk of dementia: A systematic review and meta-analysis of longitudinal cohort studies. Ageing Res. Rev..

[4] Lam J.A., Murray E.R., Yu K.E., Ramsey M., Nguyen T.T., Mishra J., Martis B., Thomas M.L., Lee E.E. (2021). Neurobiology of loneliness: A systematic review. Neuropsychopharmacology.

[5] Cole S.W., Hawkley L.C., Arevalo J.M., Sung C.Y., Rose R.M., Cacioppo J.T. (2007). Social regulation of gene expression in human leukocytes. Genome Biol..

[6] Cole S.W., Hawkley L.C., Arevalo J.M.G., Cacioppo J.T. (2011). Transcript origin analysis identifies antigen-presenting cells as primary targets of socially regulated gene expression in leukocytes. Proc. Natl. Acad. Sci. USA.

[7] Canli T., Wen R., Wang X., Mikhailik A., Yu L., Fleischman D., Wilson R.S., Bennett D.A. (2016). Differential transcriptome expression in human nucleus accumbens as a function of loneliness. Mol. Psychiatry.

[8] Rilling J.K., Gutman D.A., Zeh T.R., Pagnoni G., Berns G.S., Kilts C.D. (2002). A Neural Basis for Social Cooperation. Neuron.

[9] Davey C.G., Allen N.B., Harrison B.J., Dwyer D.B., Yücel M. (2009). Being liked activates primary reward and midline self-related brain regions. Hum. Brain Mapp..

[10] Paci P., Colombo T., Fiscon G., Gurtner A., Pavesi G., Farina L. (2017). SWIM: A computational tool to unveiling crucial nodes in complex biological networks. Sci. Rep..

[11] Fiscon G., Conte F., Farina L., Paci P. (2018). Network-Based Approaches to Explore Complex Biological Systems towards Network Medicine. Genes.

[12] Zhang B., Horvath S. (2005). A General Framework for Weighted Gene Co-Expression Network Analysis. Stat. Appl. Genet. Mol. Biol..

[13] Paci P., Fiscon G., Conte F., Licursi V., Morrow J., Hersh C., Cho M., Castaldi P., Glass K., Silverman E.K. (2020). Integrated transcriptomic correlation network analysis identifies COPD molecular determinants. Sci. Rep..

[14] Potashkin J.A., Bottero V., Santiago J.A., Quinn J.P. (2019). Computational identification of key genes that may regulate gene expression reprogramming in Alzheimer’s patients. PLoS ONE.

[15] Potashkin J.A., Bottero V., Santiago J.A., Quinn J.P. (2020). Bioinformatic Analysis Reveals Phosphodiesterase 4D-Interacting Protein as a Key Frontal Cortex Dementia Switch Gene. Int. J. Mol. Sci..

[16] Bottero V., Powers D., Yalamanchi A., Quinn J., Potashkin J. (2021). Key Disease Mechanisms Linked to Alzheimer’s Disease in the Entorhinal Cortex. Int. J. Mol. Sci..

[17] Santiago J., Quinn J., Potashkin J. (2021). Network Analysis Identifies Sex-Specific Gene Expression Changes in Blood of Amyotrophic Lateral Sclerosis Patients. Int. J. Mol. Sci..

[18] Bottero V., Santiago J.A., Quinn J.P., Potashkin J.A. (2022). Key Disease Mechanisms Linked to Amyotrophic Lateral Sclerosis in Spinal Cord Motor Neurons. Front. Mol. Neurosci..

[19] Santiago J.A., Quinn J.P., Potashkin J.A. (2022). Physical Activity Rewires the Human Brain against Neurodegeneration. Int. J. Mol. Sci..

[20] Fiscon G., Conte F., Licursi V., Nasi S., Paci P. (2018). Computational identification of specific genes for glioblastoma stem-like cells identity. Sci. Rep..

[21] Fiscon G., Conte F., Paci P. (2018). SWIM tool application to expression data of glioblastoma stem-like cell lines, corresponding primary tumors and conventional glioma cell lines. BMC Bioinform..

[22] Long J., Ray B., Lahiri D.K. (2014). MicroRNA-339-5p Down-regulates Protein Expression of β-Site Amyloid Precursor Protein-Cleaving Enzyme 1 (BACE1) in Human Primary Brain Cultures and Is Reduced in Brain Tissue Specimens of Alzheimer Disease Subjects. J. Biol. Chem..

[23] Pircs K., Petri R., Madsen S., Brattås P.L., Vuono R., Ottosson D.R., St-Amour I., Hersbach B., Matusiak-Brückner M., Lundh S.H. (2018). Huntingtin Aggregation Impairs Autophagy, Leading to Argonaute-2 Accumulation and Global MicroRNA Dysregulation. Cell Rep..

[24] Patel D., Zhang X., Farrell J., Lunetta K., Farrer L. (2021). Set-Based Rare Variant Expression Quantitative Trait Loci in Blood and Brain from Alzheimer Disease Study Participants. Genes.

[25] Hampel H., Caraci F., Cuello A.C., Caruso G., Nisticò R., Corbo M., Baldacci F., Toschi N., Garaci F., Chiesa P.A. (2020). A Path Toward Precision Medicine for Neuroinflammatory Mechanisms in Alzheimer’s Disease. Front. Immunol..

[26] Eyre H.A., Eskin A., Nelson S.F., St Cyr N.M., Siddarth P., Baune B.T., Lavretsky H. (2015). Genomic predictors of remission to antidepressant treatment in geriatric depression using genome-wide expression analyses: A pilot study. Int. J. Geriatr. Psychiatry.

[27] Gusev F.E., Reshetov D.A., Mitchell A.C., Andreeva T.V., Dincer A., Grigorenko A.P., Fedonin G., Halene T., Aliseychik M., Filippova E. (2019). Chromatin profiling of cortical neurons identifies individual epigenetic signatures in schizophrenia. Transl. Psychiatry.

[28] Berger M., Cooter M., Roesler A.S., Chung S., Park J., Modliszewski J.L., VanDusen K.W., Thompson J.W., Moseley A., Devinney M.J. (2021). APOE4 Copy Number-Dependent Proteomic Changes in the Cerebrospinal Fluid. J. Alzheimer’s Dis..

[29] Smyth L.C.D., Murray H.C., Hill M., van Leeuwen E., Highet B., Magon N.J., Osanlouy M., Mathiesen S.N., Mockett B., Singh-Bains M.K. (2022). Neutrophil-vascular interactions drive myeloperoxidase accumulation in the brain in Alzheimer’s disease. Acta Neuropathol. Commun..

[30] Katzeff J.S., Bright F., Lo K., Kril J.J., Connolly A., Crossett B., Ittner L.M., Kassiou M., Loy C.T., Hodges J.R. (2020). Altered serum protein levels in frontotemporal dementia and amyotrophic lateral sclerosis indicate calcium and immunity dysregulation. Sci. Rep..

[31] Lodeiro M., Puerta E., Ismail M.-A., Rodriguez-Rodriguez P., Rönnbäck A., Codita A., Parrado-Fernandez C., Maioli S., Gil-Bea F., Merino-Serrais P. (2016). Aggregation of the Inflammatory S100A8 Precedes Aβ Plaque Formation in Transgenic APP Mice: Positive Feedback for S100A8 and Aβ Productions. J. Gerontol. Ser. A.

[32] Rajkumar A.P., Bidkhori G., Shoaie S., Clarke E., Morrin H., Hye A., Williams G., Ballard C., Francis P.T., Aarsland D. (2019). Postmortem Cortical Transcriptomics of Lewy Body Dementia Reveal Mitochondrial Dysfunction and Lack of Neuroinflammation. Am. J. Geriatr. Psychiatry.

[33] Perez-Nievas B.G., Johnson L., Beltran-Lobo P., Hughes M.M., Gammallieri L., Tarsitano F., Myszczynska M.A., Vazquez-Villasenor I., Jimenez-Sanchez M., Troakes C. (2021). Astrocytic C–X–C motif chemokine ligand-1 mediates β-amyloid-induced synaptotoxicity. J. Neuroinflamm..

[34] Rezazadeh M., Gharesouran J., Movafagh A., Taheri M., Darvish H., Emamalizadeh B., Shahmohammadibeni N., Khorshid H.R.K., Behmanesh M., Sahraian M.A. (2015). Dominant and Protective Role of the CYTH4 Primate-Specific GTTT-Repeat Longer Alleles Against Neurodegeneration. J. Mol. Neurosci..

[35] Khademi E., Alehabib E., Shandiz E.E., Ahmadifard A., Andarva M., Jamshidi J., Rahimi-Aliabadi S., Pouriran R., Nejad F.R., Mansoori N. (2017). Support for “Disease-Only” Genotypes and Excess of Homozygosity at the CYTH4 Primate-Specific GTTT-Repeat in Schizophrenia. Genet. Test. Mol. Biomarkers.

[36] Dai D., Yuan J., Wang Y., Xu J., Mao C., Xiao Y. (2019). Peli1 controls the survival of dopaminergic neurons through modulating microglia-mediated neuroinflammation. Sci. Rep..

[37] Young J., Gallagher E., Koska K., Guetta-Baranes T., Morgan K., Thomas A., Brookes K.J. (2021). Genome-wide association findings from the brains for dementia research cohort. Neurobiol. Aging.

[38] Yin P., Xue Y., Wang T., Zhong D., Li G. (2021). The Therapeutic Targets of Fingolimod (FTY720) Are Involved in Pathological Processes in the Frontal Cortex of Alzheimer’s Disease Patients: A Network Pharmacology Study. Front. Aging Neurosci..

[39] Rossi J.J., Rosenfeld J.A., Chan K.M., Streff H., Nankivell V., Peet D.J., Whitelaw M.L., Bersten D.C. (2021). Molecular characterisation of rare loss-of-function NPAS3 and NPAS4 variants identified in individuals with neurodevelopmental disorders. Sci. Rep..

[40] Bradshaw N.J., Korth C. (2018). Protein misassembly and aggregation as potential convergence points for non-genetic causes of chronic mental illness. Mol. Psychiatry.

[41] Michaelson J.J., Shin M.-K., Koh J.-Y., Brueggeman L., Zhang A., Katzman A., McDaniel L., Fang M., Pufall M., Pieper A. (2017). Neuronal PAS Domain Proteins 1 and 3 Are Master Regulators of Neuropsychiatric Risk Genes. Biol. Psychiatry.

[42] Nucifora L.G., Wu Y.C., Lee B.J., Sha L., Margolis R.L., Ross C.A., Sawa A., Nucifora F.C. (2016). A Mutation in NPAS3 That Segregates with Schizophrenia in a Small Family Leads to Protein Aggregation. Complex Psychiatry.

[43] Yu L., Arbez N., Nucifora L.G., Sell G.L., Delisi L.E., Ross C.A., Margolis R.L., Nucifora F.C. (2013). A mutation in NPAS3 segregates with mental illness in a small family. Mol. Psychiatry.

[44] Sha L., MacIntyre L., Machell J.A., Kelly M.P., Porteous D.J., Brandon N.J., Muir W.J., Blackwood D.H., Watson D.G., Clapcote S.J. (2011). Transcriptional regulation of neurodevelopmental and metabolic pathways by NPAS3. Mol. Psychiatry.

[45] Diaz-Ortiz M.E., Seo Y., Posavi M., Cordon M.C., Clark E., Jain N., Charan R., Gallagher M.D., Unger T.L., Amari N. (2022). GPNMB confers risk for Parkinson’s disease through interaction with α-synuclein. Science.

[46] Haavik J., Toska K. (1998). Tyrosine hydroxylase and Parkinson’s disease. Mol. Neurobiol..

[47] Tabrez S., Jabir N.R., Shakil S., Greig N.H., Alam Q., Abuzenadah A.M., Damanhouri G.A., Kamal M.A. (2012). A Synopsis on the Role of Tyrosine Hydroxylase in Parkinson’s Disease. CNS Neurol. Disord. Drug Targets.

[48] Yang L.-B., Li R., Meri S., Rogers J., Shen Y. (2000). Deficiency of Complement Defense Protein CD59 May Contribute to Neurodegeneration in Alzheimer’s Disease. J. Neurosci..

[49] Goetzl E.J., Schwartz J.B., Abner E.L., Jicha G.A., Kapogiannis D. (2018). High complement levels in astrocyte-derived exosomes of Alzheimer disease. Ann. Neurol..

[50] Siitonen M., Börjesson-Hanson A., Pöyhönen M., Ora A., Pasanen P., Bras J., Kern S., Kern J., Andersen O., Stanescu H. (2017). Multi-infarct dementia of Swedish type is caused by a 3’UTR mutation of COL4A1. Brain.

[51] Hamilton A., Vasefi M., Vander Tuin C., McQuaid R.J., Anisman H., Ferguson S.S. (2016). Chronic Pharmacological mGluR5 Inhibition Prevents Cognitive Impairment and Reduces Pathogenesis in an Alzheimer Disease Mouse Model. Cell Rep..

[52] Abd-Elrahman K.S., Hamilton A., Hutchinson S.R., Liu F., Russell R.C., Ferguson S.S.G. (2017). mGluR5 antagonism increases autophagy and prevents disease progression in the *zQ175* mouse model of Huntington’s disease. Sci. Signal..

[53] Abd-Elrahman K.S., Hamilton A., Vasefi M., Ferguson S.S.G. (2018). Autophagy is increased following either pharmacological or genetic silencing of mGluR5 signaling in Alzheimer’s disease mouse models. Mol. Brain.

[54] Seipold L., Altmeppen H., Koudelka T., Tholey A., Kasparek P., Sedlacek R., Schweizer M., Bär J., Mikhaylova M., Glatzel M. (2018). In vivo regulation of the A disintegrin and metalloproteinase 10 (ADAM10) by the tetraspanin 15. Cell. Mol. Life Sci..

[55] Shigemizu D., Asanomi Y., Akiyama S., Mitsumori R., Niida S., Ozaki K. (2022). Whole-genome sequencing reveals novel ethnicity-specific rare variants associated with Alzheimer’s disease. Mol. Psychiatry.

[56] Pascual-Morena C., Cavero-Redondo I., Reina-Gutiérrez S., Saz-Lara A., López-Gil J.F., Martínez-Vizcaíno V. (2022). Prevalence of Neuropsychiatric Disorders in Duchenne and Becker Muscular Dystrophies: A Systematic Review and Meta-analysis. Arch. Phys. Med. Rehabil..

[57] Nouri P., Götz S., Rauser B., Irmler M., Peng C., Trümbach D., Kempny C., Lechermeier C.G., Bryniok A., Dlugos A. (2020). Dose-Dependent and Subset-Specific Regulation of Midbrain Dopaminergic Neuron Differentiation by LEF1-Mediated WNT1/b-Catenin Signaling. Front. Cell Dev. Biol..

[58] Huang Y., Skwarek-Maruszewska A., Horré K., Vandewyer E., Wolfs L., Snellinx A., Saito T., Radaelli E., Corthout N., Colombelli J. (2015). Loss of GPR3 reduces the amyloid plaque burden and improves memory in Alzheimer’s disease mouse models. Sci. Transl. Med..

[59] Barbagallo C., Di Martino M., Grasso M., Salluzzo M., Scionti F., Cosentino F., Caruso G., Barbagallo D., Di Pietro C., Ferri R. (2020). Uncharacterized RNAs in Plasma of Alzheimer’s Patients Are Associated with Cognitive Impairment and Show a Potential Diagnostic Power. Int. J. Mol. Sci..

[60] Bezerra G.A., Dobrovetsky E., Seitova A., Fedosyuk S., Dhe-Paganon S., Gruber K. (2015). Structure of human dipeptidyl peptidase 10 (DPPY): A modulator of neuronal Kv4 channels. Sci. Rep..

[61] Djurovic S., Gustafsson O., Mattingsdal M., Athanasiu L., Bjella T., Tesli M., Agartz I., Lorentzen S., Melle I., Morken G. (2010). A genome-wide association study of bipolar disorder in Norwegian individuals, followed by replication in Icelandic sample. J. Affect. Disord..

[62] Mitchell A.C., Bharadwaj R., Whittle C., Krueger W., Mirnics K., Hurd Y., Rasmussen T., Akbarian S. (2013). The Genome in Three Dimensions: A New Frontier in Human Brain Research. Biol. Psychiatry.

[63] Strickland S.L., Reddy J.S., Allen M., N’Songo A., Burgess J.D., Corda M.M., Ballard T., Wang X., Carrasquillo M.M., Biernacka J.M. (2020). *MAPT* haplotype–stratified GWAS reveals differential association for AD risk variants. Alzheimer’s Dement..

[64] Seki T., Kanagawa M., Kobayashi K., Kowa H., Yahata N., Maruyama K., Iwata N., Inoue H., Toda T. (2020). Galectin 3–binding protein suppresses amyloid-β production by modulating β-cleavage of amyloid precursor protein. J. Biol. Chem..

[65] Halbgebauer S., Nagl M., Klafki H., Haußmann U., Steinacker P., Oeckl P., Kassubek J., Pinkhardt E., Ludolph A.C., Soininen H. (2016). Modified serpinA1 as risk marker for Parkinson’s disease dementia: Analysis of baseline data. Sci. Rep..

[66] Abu-Rumeileh S., Halbgebauer S., Steinacker P., Anderl-Straub S., Polischi B., Ludolph A.C., Capellari S., Parchi P., Otto M. (2020). CSF SerpinA1 in Creutzfeldt–Jakob disease and frontotemporal lobar degeneration. Ann. Clin. Transl. Neurol..

[67] Barba L., Halbgebauer S., Paoletti F.P., Bellomo G., Abu-Rumeileh S., Steinacker P., Massa F., Parnetti L., Otto M. (2022). Specific Cerebrospinal Fluid SerpinA1 Isoform Pattern in Alzheimer’s Disease. Int. J. Mol. Sci..

[68] Zhao H., Wei J., Du Y., Chen P., Liu X., Liu H., Alzheimer’s Disease Neuroimaging Initiative (2022). Improved cognitive impairments by silencing DMP1 via enhancing the proliferation of neural progenitor cell in Alzheimer-like mice. Aging Cell.

[69] Hiew L.-F., Poon C.-H., You H.-Z., Lim L.-W. (2021). TGF-β/Smad Signalling in Neurogenesis: Implications for Neuropsychiatric Diseases. Cells.

[70] Tesseur I., Zou K., Esposito L., Bard F., Berber E., Van Can J., Lin A.H., Crews L., Tremblay P., Mathews P. (2006). Deficiency in neuronal TGF-β signaling promotes neurodegeneration and Alzheimer’s pathology. J. Clin. Investig..

[71] Dumitriu A., Latourelle J., Hadzi T.C., Pankratz N., Garza D., Miller J.P., Vance J., Foroud T., Beach T.G., Myers R. (2012). Gene Expression Profiles in Parkinson Disease Prefrontal Cortex Implicate FOXO1 and Genes under Its Transcriptional Regulation. PLoS Genet..

[72] Pardeshi R., Bolshette N., Gadhave K., Ahire A., Ahmed S., Cassano T., Gupta V.B., Lahkar M. (2017). Insulin signaling: An opportunistic target to minify the risk of Alzheimer’s disease. Psychoneuroendocrinology.

[73] Lee S., Dong H.H. (2017). FoxO integration of insulin signaling with glucose and lipid metabolism. J. Endocrinol..

[74] Santiago J.A., Bottero V., Potashkin J.A. (2019). Transcriptomic and Network Analysis Highlight the Association of Diabetes at Different Stages of Alzheimer’s Disease. Front. Neurosci..

[75] Srikanth V., Maczurek A., Phan T., Steele M., Westcott B., Juskiw D., Münch G. (2011). Advanced glycation endproducts and their receptor RAGE in Alzheimer’s disease. Neurobiol. Aging.

[76] Guo Q., Zhu X., Wei R., Zhao L., Zhang Z., Yin X., Zhang Y., Chu C., Wang B., Li X. (2020). miR-130b-3p regulates M1 macrophage polarization via targeting IRF1. J. Cell. Physiol..

[77] Chu Y.-B., Li J., Jia P., Cui J., Zhang R., Kang X., Lv M., Zhang S. (2021). Irf1- and Egr1-activated transcription plays a key role in macrophage polarization: A multiomics sequencing study with partial validation. Int. Immunopharmacol..

[78] Gao T., Jernigan J., Raza S.A., Dammer E.B., Xiao H., Seyfried N.T., Levey A.I., Rangaraju S. (2019). Transcriptional regulation of homeostatic and disease-associated-microglial genes by IRF1, LXRβ, and CEBPα. Glia.

[79] Ponnusamy M., Wang S., Yuksel M., Hansen M.T., Blazier D.M., McMillan J.D., Zhang X., Dammer E.B., Collier L., Thinakaran G. (2022). Loss of forebrain BIN1 attenuates hippocampal pathology and neuroinflammation in a tauopathy model. Brain.

[80] Sudwarts A., Ramesha S., Gao T., Ponnusamy M., Wang S., Hansen M., Kozlova A., Bitarafan S., Kumar P., Beaulieu-Abdelahad D. (2022). BIN1 is a key regulator of proinflammatory and neurodegeneration-related activation in microglia. Mol. Neurodegener..

[81] Vied C.M., Freudenberg F., Wang Y., Raposo A.A.S.F., Feng D., Nowakowski R.S. (2014). A multi-resource data integration approach: Identification of candidate genes regulating cell proliferation during neocortical development. Front. Neurosci..

[82] Knepper J.L., James A.C., Ming J.E. (2006). TGIF, a gene associated with human brain defects, regulates neuronal development. Dev. Dyn..

[83] Cui A., Fan H., Zhang Y., Zhang Y., Niu D., Liu S., Liu Q., Ma W., Shen Z., Shen L. (2019). Dexamethasone-induced Krüppel-like factor 9 expression promotes hepatic gluconeogenesis and hyperglycemia. J. Clin. Investig..

[84] Piccinin E., Sardanelli A., Seibel P., Moschetta A., Cocco T., Villani G. (2021). PGC-1s in the Spotlight with Parkinson’s Disease. Int. J. Mol. Sci..

[85] Zheng B., Liao Z., Locascio J.J., Lesniak K.A., Roderick S.S., Watt M.L., Eklund A.C., Zhang-James Y., Kim P.D., Hauser M.A. (2010). PGC-1α, A Potential Therapeutic Target for Early Intervention in Parkinson’s Disease. Sci. Transl. Med..

[86] Liu E.Y., Cali C.P., Lee E.B. (2017). RNA metabolism in neurodegenerative disease. Dis. Model. Mech..

[87] Nussbacher J.K., Tabet R., Yeo G.W., Lagier-Tourenne C. (2019). Disruption of RNA Metabolism in Neurological Diseases and Emerging Therapeutic Interventions. Neuron.

[88] Santiago J.A., Potashkin J.A. (2015). Network-based metaanalysis identifies HNF4A and PTBP1 as longitudinally dynamic biomarkers for Parkinson’s disease. Proc. Natl. Acad. Sci. USA.

[89] Vinogradov A.E., Anatskaya O.V. (2021). Growth of Biological Complexity from Prokaryotes to Hominids Reflected in the Human Genome. Int. J. Mol. Sci..

[90] Martin L.A., Neighbors H.W., Griffith D.M. (2013). The experience of symptoms of depression in men vs women: Analysis of the National Comorbidity Survey Replication. JAMA Psychiatry.

[91] Santiago J.A., Quinn J.P., Potashkin J.A. (2022). Sex-specific transcriptional rewiring in the brain of Alzheimer’s disease patients. Front Aging Neurosci..

[92] Naj A.C., Beecham G.W., Martin E.R., Gallins P.J., Powell E.H., Konidari I., Whitehead P.L., Cai G., Haroutunian V., Scott W.K. (2010). Dementia Revealed: Novel Chromosome 6 Locus for Late-Onset Alzheimer Disease Provides Genetic Evidence for Folate-Pathway Abnormalities. PLoS Genet..

[93] Logue M., Schu M., Vardarajan B.N., Buros J., Green R.C., Go R.C., Griffith P., Obisesan T.O., Shatz R., Borenstein A. (2011). A Comprehensive Genetic Association Study of Alzheimer Disease in African Americans. Arch. Neurol..

[94] Hu X., Pickering E., Liu Y.C., Hall S., Fournier H., Katz E., Dechairo B., John S., Van Eerdewegh P., Soares H. (2011). Meta-Analysis for Genome-Wide Association Study Identifies Multiple Variants at the BIN1 Locus Associated with Late-Onset Alzheimer’s Disease. PLoS ONE.

[95] Wijsman E.M., Pankratz N.D., Choi Y., Rothstein J.H., Faber K.M., Cheng R., Lee J.H., Bird T.D., Bennett D.A., Diaz-Arrastia R. (2011). Genome-Wide Association of Familial Late-Onset Alzheimer’s Disease Replicates BIN1 and CLU and Nominates CUGBP2 in Interaction with APOE. PLoS Genet..

[96] Hollingworth P., Harold D., Sims R., Gerrish A., Lambert J.-C., Carrasquillo M.M., Abraham R., Hamshere M.L., Pahwa J.S., Moskvina V. (2011). Common variants at ABCA7, MS4A6A/MS4A4E, EPHA1, CD33 and CD2AP are associated with Alzheimer’s disease. Nat. Genet..

[97] Lambert J.-C., Heath S., Even G., Campion D., Sleegers K., Hiltunen M., Combarros O., Zelenika D., Bullido M.J., Tavernier B. (2009). Genome-wide association study identifies variants at CLU and CR1 associated with Alzheimer’s disease. Nat. Genet..

[98] Antúnez C., Boada M., González-Pérez A., Gayán J., Ramírez-Lorca R., Marin J., Hernandez I., Moreno-Rey C., Morón F.J., López-Arrieta J. (2011). The membrane-spanning 4-domains, subfamily A (MS4A) gene cluster contains a common variant associated with Alzheimer’s disease. Genome Med..

[99] Harold D., Abraham R., Hollingworth P., Sims R., Gerrish A., Hamshere M.L., Pahwa J.S., Moskvina V., Dowzell K., Williams A. (2009). Genome-wide association study identifies variants at CLU and PICALM associated with Alzheimer’s disease. Nat. Genet..

[100] Martinelli-Boneschi F., Giacalone G., Magnani G., Biella G., Coppi E., Santangelo R., Brambilla P., Esposito F., Lupoli S., Clerici F. (2013). Pharmacogenomics in Alzheimer’s disease: A genome-wide association study of response to cholinesterase inhibitors. Neurobiol. Aging.

[101] Pankratz N., Beecham G.W., DeStefano A.L., Dawson T.M., Doheny K.F., Factor S.A., Hamza T.H., Hung A.Y., Hyman B.T., Ivinson A.J. (2012). Meta-analysis of Parkinson’s Disease: Identification of a novel locus, RIT2. Ann. Neurol..

[102] Nalls M.A., Pankratz N., Lill C.M., Do C.B., Hernandez D.G., Saad M., DeStefano A.L., Kara E., Bras J., Sharma M. (2014). Large-scale meta-analysis of genome-wide association data identifies six new risk loci for Parkinson’s disease. Nat. Genet..

[103] Chang D., Nalls M.A., Hallgrímsdóttir I.B., Hunkapiller J., Van Der Brug M., Cai F., Kerchner G.A., Ayalon G., International Parkinson’s Disease Genomics Consortium, 23andMe Research Team (2017). A meta-analysis of genome-wide association studies identifies 17 new Parkinson’s disease risk loci. Nat. Genet..

[104] Simón-Sánchez J., Schulte C., Bras J.M., Sharma M., Gibbs J.R., Berg D., Paisan-Ruiz C., Lichtner P., Scholz S.W., Hernandez D.G. (2009). Genome-wide association study reveals genetic risk underlying Parkinson’s disease. Nat. Genet..

[105] Lee S.L., Pearce E., Ajnakina O., Johnson S., Lewis G., Mann F., Pitman A., Solmi F., Sommerlad A., Steptoe A. (2020). The association between loneliness and depressive symptoms among adults aged 50 years and older: A 12-year population-based cohort study. Lancet Psychiatry.

[106] Shyn S.I., Shi J., Kraft J.B., Potash J.B., Knowles J.A., Weissman M.M., Garriock H.A., Yokoyama J.S., McGrath P.J., Peters E.J. (2009). Novel loci for major depression identified by genome-wide association study of Sequenced Treatment Alternatives to Relieve Depression and meta-analysis of three studies. Mol. Psychiatry.

[107] Wray N.R., Pergadia M.L., Blackwood D.H.R., Penninx B.W.J.H., Gordon S.D., Nyholt D.R., Ripke S., MacIntyre D.J., A McGhee K., Maclean A.W. (2010). Genome-wide association study of major depressive disorder: New results, meta-analysis, and lessons learned. Mol. Psychiatry.

[108] Garriock H.A., Kraft J.B., Shyn S.I., Peters E.J., Yokoyama J.S., Jenkins G.D., Reinalda M.S., Slager S.L., McGrath P.J., Hamilton S.P. (2010). A Genomewide Association Study of Citalopram Response in Major Depressive Disorder. Biol. Psychiatry.

[109] Ikeda M., Aleksic B., Kinoshita Y., Okochi T., Kawashima K., Kushima I., Ito Y., Nakamura Y., Kishi T., Okumura T. (2011). Genome-Wide Association Study of Schizophrenia in a Japanese Population. Biol. Psychiatry.

[110] O’Donovan M.C., Craddock N., Norton N., Williams H., Peirce T., Moskvina V., Nikolov I., Hamshere M., Carroll L., Georgieva L. (2008). Identification of loci associated with schizophrenia by genome-wide association and follow-up. Nat. Genet..

[111] Shi J., Levinson D.F., Duan J., Sanders A.R., Zheng Y., Pe’Er I., Dudbridge F., Holmans P.A., Whittemore A.S., Mowry B.J. (2009). Common variants on chromosome 6p22.1 are associated with schizophrenia. Nature.

[112] The Schizophrenia Psychiatric Genome-Wide Association Study (GWAS) Consortium (2011). Genome-wide association study identifies five new schizophrenia loci. Nat. Genet..

[113] Contreras A., Valiente C., Vázquez C., Trucharte A., Peinado V., Varese F., Bentall R.P. (2022). The network structure of paranoia dimensions and its mental health correlates in the general population: The core role of loneliness. Schizophr. Res..

[114] Ma X., Deng W., Liu X., Li M., Chen Z., He Z., Wang Y., Wang Q., Hu X., Collier D.A. (2011). A genome-wide association study for quantitative traits in schizophrenia in China. Genes Brain Behav..

[115] Steen O.D., Ori A.P.S., Wardenaar K.J., van Loo H.M. (2022). Loneliness associates strongly with anxiety and depression during the COVID pandemic, especially in men and younger adults. Sci. Rep..

[116] Czeisler M.É., Lane R.I., Petrosky E., Wiley J.F., Christensen A., Njai R., Weaver M.D., Robbins R., Facer-Childs E.R., Barger L.K. (2020). Mental Health, Substance Use, and Suicidal Ideation During the COVID-19 Pandemic—United States, June 24–30, 2020. MMWR Morb. Mortal. Wkly. Rep..

[117] Chételat G., Lutz A., Arenaza-Urquijo E., Collette F., Klimecki O., Marchant N. (2018). Why could meditation practice help promote mental health and well-being in aging?. Alzheimer’s Res. Ther..

[118] Lazar S.W., Kerr C.E., Wasserman R.H., Gray J.R., Greve D.N., Treadway M.T., McGarvey M., Quinn B.T., Dusek J.A., Benson H. (2005). Meditation experience is associated with increased cortical thickness. Neuroreport.

[119] Memon A.A., Coleman J.J., Amara A.W. (2020). Effects of exercise on sleep in neurodegenerative disease. Neurobiol. Dis..

[120] Mamalaki E., Ntanasi E., Hatzimanolis A., Basta M., Kosmidis M.H., Dardiotis E., Hadjigeorgiou G.M., Sakka P., Scarmeas N., Yannakoulia M. (2023). The Association of Adherence to the Mediterranean Diet with Depression in Older Adults Longitudinally Taking into Account Cognitive Status: Results from the HELIAD Study. Nutrients.

[121] Zhou G., Soufan O., Ewald J., Hancock R.E.W., Basu N., Xia J. (2019). NetworkAnalyst 3.0: A visual analytics platform for comprehensive gene expression profiling and meta-analysis. Nucleic Acids Res..

[122] Kupershmidt I., Su Q.J., Grewal A., Sundaresh S., Halperin I., Flynn J., Shekar M., Wang H., Park J., Cui W. (2010). Ontology-Based Meta-Analysis of Global Collections of High-Throughput Public Data. PLoS ONE.

