# Supplementary figures and images for: Co-Expression Network Analysis Identifies Molecular Determinants of Loneliness Associated with Neuropsychiatric and Neurodegenerative Diseases
Source: Int J Mol Sci. 2023 Mar 21;24(6):5909. doi: 10.3390/ijms24065909 (PMC10058494; doi:10.3390/ijms24065909)

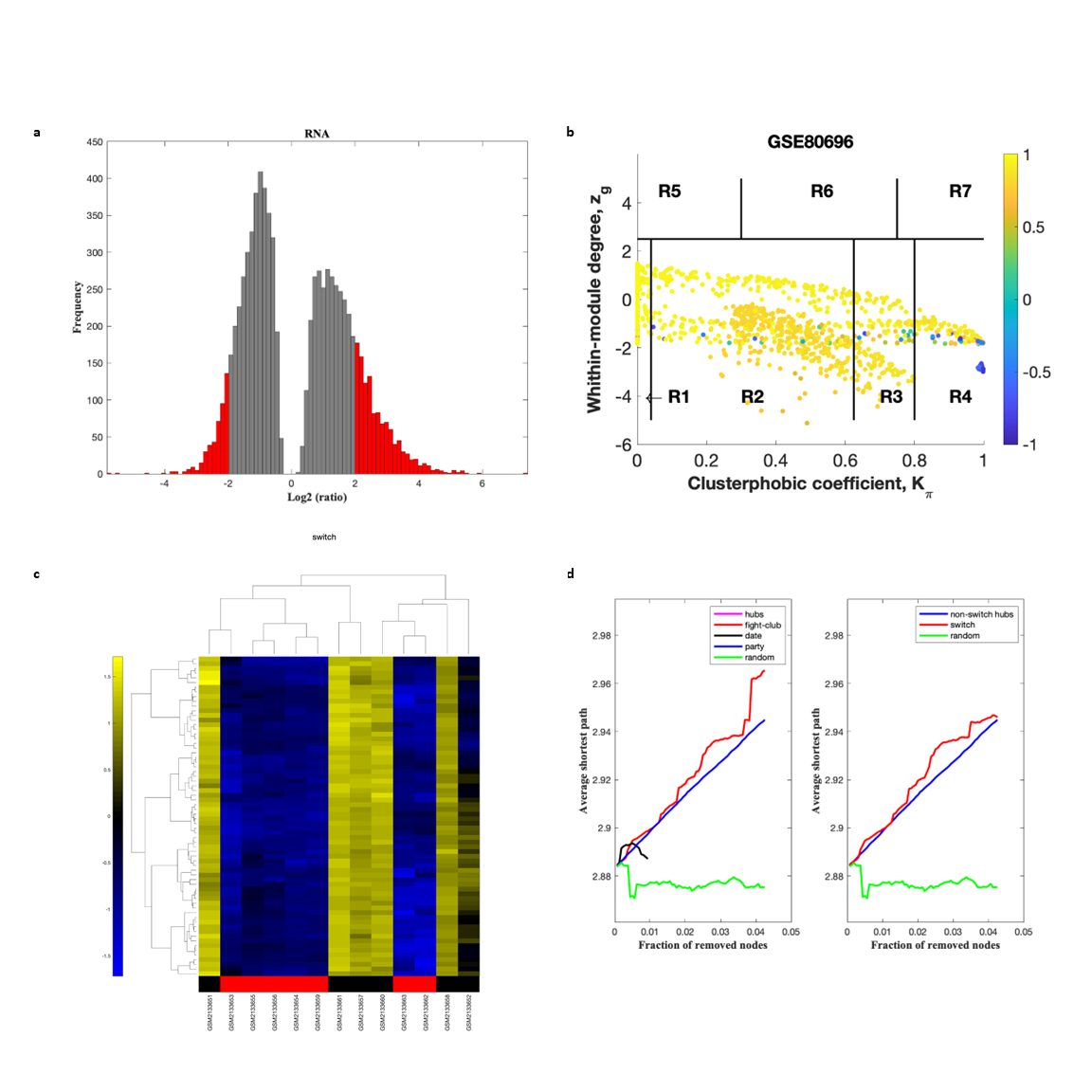

Supplement: Supplementary file 1 [file ijms-24-05909-s001.zip › Santiago_Supplementaty_Fig. S1.tif]
